# Comparison of long-term outcomes after trans-catheter aortic valve implantation between patients primarily diagnosed by cardiac murmur and those diagnosed by other reasons

**DOI:** 10.1371/journal.pone.0247588

**Published:** 2021-02-19

**Authors:** Yousuke Taniguchi, Kenichi Sakakura, Yohei Nomura, Masashi Hatori, Kaho Shibata, Yusuke Tamanaha, Taku Kasahara, Takunori Tsukui, Tatsuro Ibe, Kei Yamamoto, Hiroyuki Jinnouchi, Hiroshi Wada, Atsushi Yamaguchi, Hideo Fujita

**Affiliations:** 1 Division of Cardiovascular Medicine, Saitama Medical Center, Jichi Medical University, Saitama, Japan; 2 Department of Cardiovascular Surgery, Saitama Medical Center, Jichi Medical University, Saitama, Japan; Azienda Ospedaliero Universitaria Careggi, ITALY

## Abstract

Careful auscultation is the first step to diagnose aortic stenosis (AS). The aim of this study was to compare clinical outcomes following transcatheter aortic valve implantation (TAVI) between the patients primarily diagnosed by heart murmur and those diagnosed by other reasons. We retrospectively included 258 patients who underwent TAVI in our medical center, and divided those into the murmur group (n = 81) and the other-reason group (n = 177) according to the primary reason for AS diagnosis. The primary endpoint was the major adverse cardiovascular and cerebrovascular events (MACCE), which was defined as the composite of cardiovascular death, hospitalization due to acute decompensated heart failure, and disabling stroke. The murmur group included younger patients than the other-reason group (82.8 year-old vs. 84.0 year-old, P = 0.02). History of AF was more frequently observed in the other-reason group than in the murmur group (21.5% vs. 7.4%, P <0.01). STS score and logistic EuroSCORE were lower in the murmur group than in the other-reason group (STS: 4.7% vs. 7.2%, P <0.01, logistic EuroSCORE: 8.3% vs. 11.2%, P <0.01). The median follow-up period was 562 days. MACCE was more frequently observed in the other-reason group than in the murmur group (27.7% vs. 9.9%, Log Rank P <0.01). The multivariate COX hazard analysis revealed that the AS patients primarily diagnosed by heart murmur was inversely associated with MACCE (HR 0.38, 95%CI 0.17–0.86, P = 0.020). Among AS patients who underwent TAVI, the patients primarily diagnosed by heart murmur were significantly associated with favorable long-term clinical outcomes.

## Introduction

Trans-catheter aortic valve implantation (TAVI) has been a standard therapy for severe aortic valve stenosis (AS), especially in high surgical risk patients [1–4]. Frailty, lower active daily life (ADL), or malnutrition have been reported as major predictors of poor long-term outcomes after TAVI [5–9]. Moreover, symptomatic AS was associated with poor clinical outcomes [10], whereas asymptomatic AS was associated with favorable clinical outcomes [11]. Thus, it is important to detect severe AS when patients are asymptomatic.

On the other hand, it is difficult to find asymptomatic patients with severe AS, partly because routine medical checkups do not include echocardiography. Careful auscultation by primary care physician is a key to find asymptomatic patients with severe AS. We hypothesized that severe AS patients primarily diagnosed by heart murmur would have better clinical outcomes as compared to severe AS patients detected by other reasons. The aim of this study was to compare clinical outcomes following TAVI between the patients primarily diagnosed by heart murmur and the patients diagnosed by other reasons.

## Materials and methods

### Study design

We screened consecutive patients who underwent TAVI in our medical center between July 2014 and December 2019. Indications and procedural strategies of each TAVI were discussed in a weekly heart team conference in our medical center. All patients that underwent TAVI in our center had some symptoms, at least mild symptoms. Inclusion criteria was the patients who underwent TAVI in our medical center during the above study period. Exclusion criteria was the absence of medical records regarding the primary reason for AS diagnosis. We retrospectively investigated each patient’s primary reason for AS diagnosis from their medical records. The final study population was divided into the two groups according to each primary reason for AS diagnosis. The patients whose primary reason for AS diagnosis was “heart murmur” were defined as “the murmur group”, and the other patients were defined as “the other-reason group”.

The primary endpoint of this study was major adverse cardiovascular and cerebrovascular events (MACCE), which was defined as the composite of cardiovascular death, hospitalization due to acute decompensated heart failure (ADHF), and disabling stroke. Cardiovascular death and disabling stroke were defined according to the criteria of Valve Academic Research Consortium 2 (VARC2) [12]. We set the procedure date as the index day, and the final study date as June 30^th^, 2020. Most patients were followed up by yearly outpatient clinic until five years after TAVI, and when a patient did not come to the outpatient clinic, we followed up by phone. This study was approved by the Institutional Review Board of Saitama Medical Center, Jichi Medical University (S20-138), and written informed consent was waived because of the retrospective study design.

### TAVI procedure

In our medical center, 80.6% of TAVI were performed by trans-femoral approach (TF). TF-TAVI in our medical center was described in elsewhere [9,13]. In addition, 16.1% of TAVI were performed by trans-apical approach (TA), and other cases were by trans-iliac, direct aorta or trans subclavian approaches (3.3%). During TA-TAVI, valve crossing was achieved by a J-shaped conventional wire (0.035 inch), and then the conventional wire was exchanged to a stiff wire. After wire crossing, we inserted 18- or 21-Fr original sheaths for TA-TAVI, and implanted a balloon expandable bioprosthetic valve. Trans-iliac, direct aorta, or trans subclavian approach TAVI were performed in accordance with TF-TAVI.

In our institution, the protocol of anti-platelet or anti-coagulant therapy after TAVI were generally dual anti-platelet therapy (DAPT) for 3–6 months. After DAPT, permanent single anti-platelet therapy (SAPT) was recommended. However, these protocols were modified according to the patient’s conditions such as presence of AF. For example, if a patient had AF, direct oral anti-coagulant (DOAC) and SAPT might be prescribed.

### Definitions

Hypertension was defined as receiving treatment for hypertension before admission [9]. Dyslipidemia was defined as total cholesterol >220 mg/dL, low-density lipoprotein cholesterol >140 mg/dL, or medical treatment for dyslipidemia before admission [14]. Diabetes mellitus (DM) was defined as hemoglobin A1c (HbA1c) >6.5% (national glycol-hemoglobin standardization program (NGSP) value) or medical treatment for DM [15]. Estimated glomerular filtration rate (eGFR) was calculated by modification of diet in renal disease (MDRD) method adjusted for Japanese population [16,17]. Anemia was determined by world health organization (WHO) criteria: hemoglobin value <13 g/dL in males and <12 g/dL in females [18]. Frailty was evaluated according to Clinical Frailty Scale (CFS) by medical professionals who cared the patients directly before TAVI [19]. Each STS score and logistic EuroSCORE were calculated before procedure using with online available calculators [20–23]. History of atrial fibrillation (AF) was defined as receiving treatment for AF, or document on electrocardiogram (ECG) or monitoring before procedure. Atrio-ventricular block (AV block) was defined as any degrees of AV block on ECG or monitoring before procedure, and the first degree AV block was defined as that of PR interval >200 ms [24]. Echocardiography recorded that the left ventricular ejection fraction (modified Simpson method), aortic valve peak velocity and mean pressure gradient. Aortic valve area was calculated by equation of continuity from those data. Echocardiography also recorded the severity of aortic valve insufficiency according to the reached distance of regurgitant color jet and that of mitral valve insufficiency according to the percentage of jet area. Pulmonary hypertension (PH) was defined as the pressure gradient of tri-cuspid valve regurgitation ≥30 mmHg. Low-flow low-gradient AS was defined as the combination of peak velocity of aortic valve <4.0 m/s, aortic valve area ≤1.0 cm^2^, and stroke volume index ≤35 ml/m^2^ according to the guideline 2017 of European society of cardiology [25]. Screening contrast-enhanced computed tomography (CT) was performed for anatomical characteristics of aortic valve, Valsalva sinus, sino-tubular junction and access information. The complications related to TAVI such as conversion to open surgery, coronary obstruction or ventricular perforation were defined according to the VARC2 criteria [12].

### Statistical methods

Categorical data were presented as number and percentage, and continuous data were presented as mean ± standard deviation (SD). Normally distributed continuous variables were compared using an unpaired Student *t*-test. Other continuous variables were compared using a Mann-Whitney *U*-test. Categorical data were compared using the chi-square test or Fisher’s exact test. The Kaplan-Meyer survival curves were constructed, and the difference between the two survival curves was compared by a Log Rank test. We also performed multivariate COX hazard analysis with likelihood ratio statistical criteria using backward elimination method to confirm the association between the murmur group and MACCE after controlling confounding factors. In this model, MACCE was used as a dependent variable. We adopted marginally significant variables (P <0.10) in univariate comparisons as independent variables. Variables that had missing values were not included in the multivariate analysis. Hazard ratios (HR) and 95% confidence intervals (95% CI) were calculated. P value <0.05 was considered statistically significant. All analyses were performed using statistical software, SPSS PASW Statistics 25, release 25.0.0 /Windows (IBM corp.).

## Results

**Fig 1:**
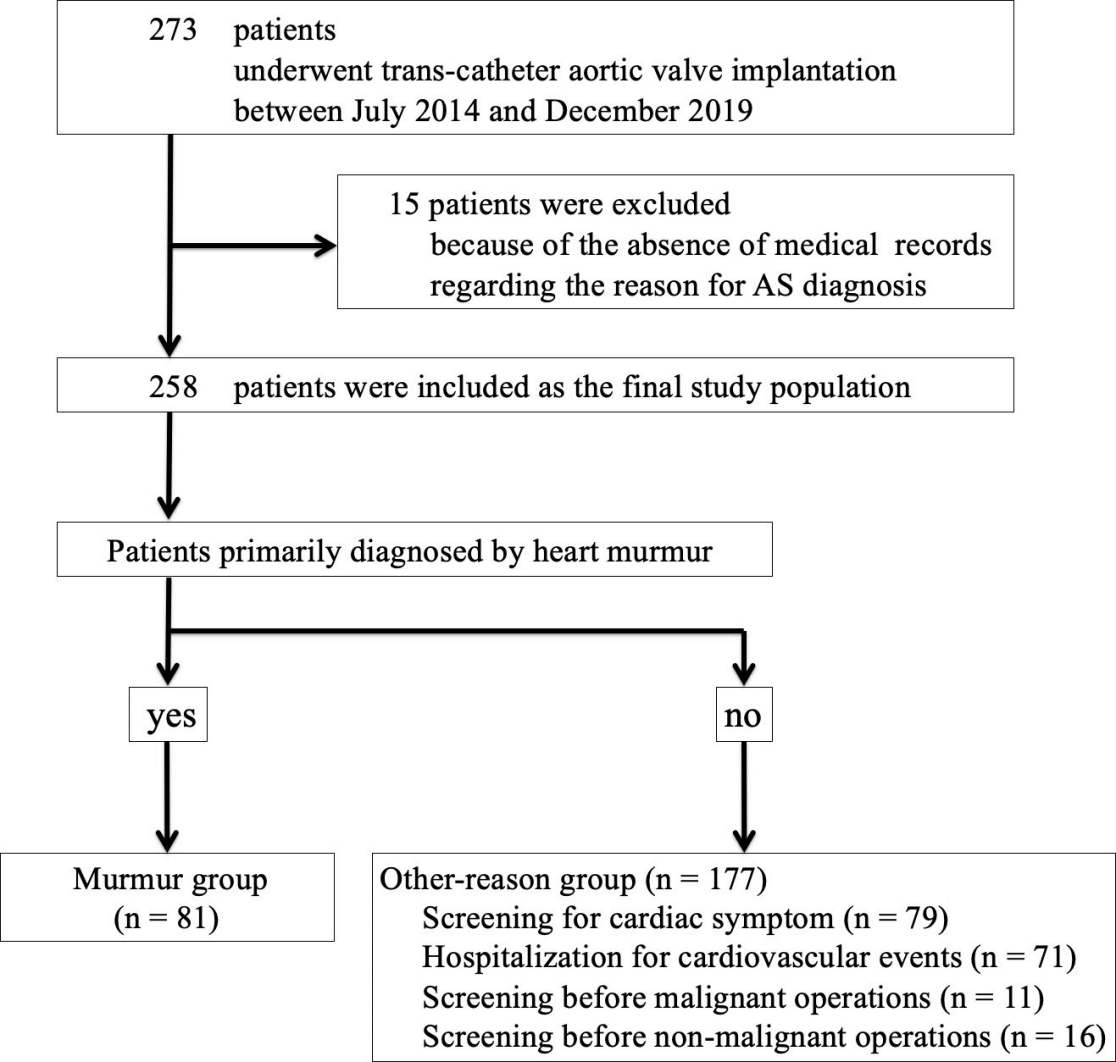
Study flowchart. AS, aortic valve stenosis.

During the study period, a total of 273 patients underwent TAVI in our medical center. Of 273 patients, 15 patients were excluded because of the absence of records regarding the primary reason for AS diagnosis. Finally, 258 patients were included for the final study population, and were divided into the murmur group (n = 81) and the other-reason group (n = 177) (Fig 1).

Table 1 shows the comparison of baseline characteristics. The murmur group included younger patients than the other-reason group (82.8 year-old vs. 84.0 year-old, P = 0.02). History of AF was more frequently observed in the other-reason group than in the murmur group (21.5% vs. 7.4%, P <0.01). Albumin value before procedure were higher in the murmur group than the other-reason group (4.1 g/dL vs. 3.8 g/dL, P <0.01). The eGFR values were also higher in the murmur group (63.2 mL/min/1.73m^2^ vs. 50.0 mL/min/1.73m^2^, P <0.01). BNP before TAVI was better in the murmur group than in the other-reason group (297 pg/mL vs. 537 pg/mL, P <0.01). Hemoglobin levels were greater in the murmur group than in the other-reason group (11.9 g/dL vs. 11.0 g/dL, p <0.01). Pulmonary hypertension was more frequently observed in the other-reason group than in the murmur group (15.3% vs. 4.9%. P = 0.02). The prevalence of diuretics users was greater in the other-reason group than in the murmur group (67.8% vs. 39.5%, P <0.01). Both STS score and logistic EuroSCORE were lower in the murmur group than in the other-reason group (STS: 4.7% vs. 7.2%, P <0.01, logistic EuroSCORE: 8.3% vs. 11.2%, P <0.01). Table 2 shows the comparison of TAVI procedures and complications between the two groups. There were no significant differences in TAVI procedures and complications. The incidence of ECMO use during TAVI was only 4.3%.

**Table 1:** Comparison of baseline characteristics.

	Overall (N = 258)	Murmur group (n = 81)	Other-reason group (n = 177)	P value
Age (year-old)	83.6 ± 5.2	82.8 ± 4.2	84.0 ± 5.6	0.02
Female gender (no.) (%)	168 (65.1)	56 (69.1)	112 (63.3)	0.40
Body surface area (m^2^)	1.46 ± 0.18	1.48 ± 0.18	1.45 ± 0.18	0.12
Smoking (no.) (%)	58 (22.7)	17 (21.5)	41 (23.2)	0.87
Hypertension (no.) (%)	211 (81.8)	69 (85.2)	142 (80.2)	0.39
Dyslipidemia (no.) (%)	138 (53.5)	50 (61.7)	88 (49.7)	0.08
Diabetes mellitus (no.) (%)	69 (26.7)	23 (28.4)	46 (26.0)	0.76
History of atrial fibrillation (no.) (%)	44 (17.1)	6 (7.4)	38 (21.5)	<0.01
Coronary artery disease (no.) (%)	84 (32.6)	21 (25.9)	63 (35.6)	0.15
Old cerebral infarction (no.) (%)	18 (7.0)	3 (3.7)	15 (8.5)	0.20
Interstitial Pneumonia (no.) (%)	21 (8.1)	4 (4.9)	17 (9.6)	0.23
Malignant diseases (no.) (%)	18 (7.0)	3 (3.7)	15 (8.5)	0.20
Electrocardiogram				
Atrio-ventricular block (no.) (%)	28 (10.9)	4 (4.9)	24 (13.6)	0.05
Right bundle branch block (no.) (%)	36 (14.0)	10 (12.3)	26 (14.7)	0.70
Laboratory data				
Albumin (g/dL)	3.9 ± 0.5	4.1 ± 0.3	3.8 ± 0.5	<0.01
eGFR (mL/min/1.73m^2^)	54.2 ± 22.2	63.2 ± 20.6	50.0 ± 21.7	<0.01
Hemoglobin (g/dL)	11.3 ± 1.6	11.9 ± 1.6	11.0 ± 1.6	<0.01
BNP (pg/mL) (n = 243)	461 ± 733	297 ± 481	537 ± 815	<0.01
Echocardiogram				
LVEF (Simpson) (n = 139) (%)	61 ± 13	61 ± 14	60 ± 13	0.44
Peak velocity of aortic valve (m/s)	4.76 ± 0.74	4.75 ± 0.70	4.76 ± 0.76	0.73
Low-flow low-gradient AS (%)	8 (3.1)	2 (2.5)	6 (3.4)	1.00
Pulmonary hypertension (no.) (%)	31 (12.0)	4 (4.9)	27 (15.3)	0.02
Computed tomography				
Annulus area (mm^2^)	404 ± 68	397 ± 61	407 ± 70	0.41
Perimeter (mm) (n = 203)	73.4 ± 6.1	72.4 ± 5.6	73.8 ± 6.3	0.20
Medications at admission (no.) (%)				
Aspirin	99 (38.4)	29 (35.8)	70 (39.5)	0.58
P2Y_12_ inhibitors	47 (18.2)	11 (13.6)	36 (20.3)	0.23
Oral anti-coagulants	29 (11.2)	5 (6.2)	24 (13.6)	0.09
Statins	133 (51.6)	46 (56.8)	87 (49.2)	0.28
ACE inhibitors or ARB	135 (52.3)	39 (48.1)	96 (54.2)	0.42
β blockers	89 (34.5)	25 (30.9)	64 (36.2)	0.48
Diuretics	152 (58.9)	32 (39.5)	120 (67.8)	<0.01
Oral hypoglycemic agents	45 (17.4)	13 (16.0)	32 (18.1)	0.73
Insulin user	9 (3.5)	3 (3.7)	6 (3.4)	1.00
Steroids	16 (6.2)	7 (8.6)	9 (5.1)	0.28
Catecholamine	2 (0.8)	0 (0)	2 (1.1)	1.00
Clinical frailty scale before procedure				0.22
1–3	132 (51.2)	47 (58.0)	85 (48.0)	
4–6	77 (29.8)	23 (28.4)	54 (30.5)	
7–9	49 (19.0)	11 (13.6)	38 (21.5)	
STS score (%)	6.4 ± 5.5	4.7 ± 2.3	7.2 ± 6.4	<0.01
logistic EuroSCORE (%) (n = 257)	10.3 ± 6.9	8.3 ± 4.7	11.2 ± 7.6	<0.01
Duration from the date of diagnosis to the date of TAVI (days) (n = 225)	666 ± 900	962 ± 1045	509 ± 772	<0.01

eGFR, estimated glomerular filtration rate; BNP, brain natriuretic peptide; LVEF, left ventricular ejection fraction; AS, aortic valve stenosis; ACE, Angiotensin converting enzyme; ARB, Angiotensin II receptor blockers; STS, society of thoracic surgeons; TAVI, transcatheter aortic valve implantation.

**Table 2:** Comparison of procedure and complications.

	Overall (N = 258)	Murmur group (n = 81)	Other-reason group (n = 177)	P value
Approach (no.) (%)				0.19
Trans femoral	210 (81.4)	71 (87.7)	139 (78.5)	
Trans apical	41 (15.9)	8 (9.9)	33 (18.6)	
Alternative	7 (2.7)	2 (2.5)	5 (2.8)	
Devices (no.) (%) Balloon expandable				0.58
SapienXT	61 (23.6)	18 (22.2)	43 (24.3)	
Sapien3	134 (51.9)	45 (55.6)	89 (50.3)	
Self expandable				
Corevalve	13 (5.0)	2 (2.5)	11 (6.2)	
Evolut series	50 (19.4)	16 (19.8)	34 (19.2)	
Size (no.) (%)				0.66
20 mm	11 (4.3)	4 (4.9)	7 (4.0)	
23 mm	120 (46.5)	41 (50.6)	79 (44.6)	
26 mm	93 (36.0)	28 (34.6)	65 (36.7)	
29 mm	34 (13.2)	8 (9.9)	26 (14.7)	
Balloon aortic valvuloplasty (no.)	163 (63.2)	50 (61.7)	113 (63.8)	0.78
Post dilatation (no.)	34 (13.2)	13 (16.0)	21 (11.9)	0.43
Operation time (min)	144 ± 64	140 ± 51	146 ± 70	0.53
Exposure time (min)	39 ± 16	42 ± 16	38 ± 16	0.07
Contrast volume (mL)	144 ± 62	147 ± 60	143 ± 63	0.38
Complications (no.) (%)	39 (15.1)	15 (18.5)	24 (13.6)	0.35
Convert to emergent open surgery	3 (1.2)	2 (2.5)	1 (0.6)	0.23
ECMO during procedure	11 (4.3)	4 (4.9)	7 (4.0)	0.75
Acute coronary obstruction	6 (2.3)	1 (1.2)	5 (2.8)	0.67
Disabling acute cerebral infarction	3 (1.2)	0 (0)	3 (1.7)	0.55
New pacemaker implantation	21 (8.1)	5 (6.2)	16 (9.0)	0.62

ECMO, Extracorporeal membrane oxygenation.

**Fig 2:**
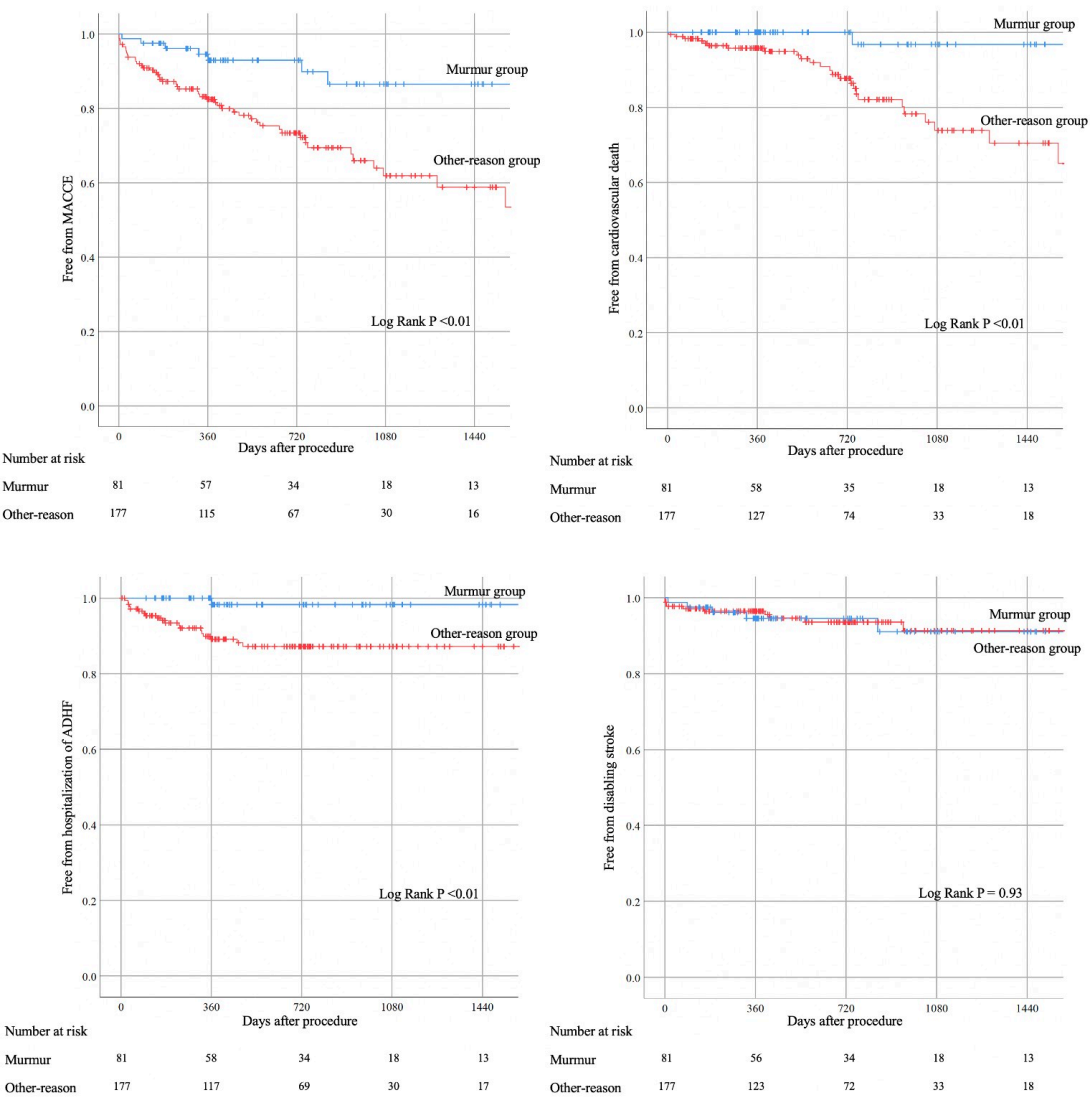
Kaplan-Meyer survival curve analyses. MACCE, major adverse cardiovascular or cerebrovascular events: Composite of cardiovascular death, history of hospitalization of acute de-compensated heart failure (ADHF), and disabling stroke.

Table 3 shows in-hospital and long-term outcomes after TAVI. Long-term outcomes were better in the murmur group than in the other-reason group with regard to MACCE (9.9% vs. 27.7%, P <0.01), cardiovascular death (1.2% vs. 14.7%, P <0.01), and hospitalization of ADHF (2.5% vs. 11.3%, P = 0.02). Fig 2 shows Kaplan-Meyer curves regarding long-term outcomes. The median follow-up period was 562 days (349–952 days). MACCE was more frequently observed in the other-reason group than in the murmur group (9.9% vs. 27.7%, P <0.01).

**Table 3:** In Hospital and long-term outcomes after TAVI.

	Overall (N = 258)	Murmur group (n = 81)	Other-reason group (n = 177)	P value
Echocardiogram after TAVI				
LVEF (Simpson) (n = 134) (%)	59 ± 13	59 ± 14	59 ± 12	0.04
Aortic valve				
Peak velocity (m/s) (n = 257)	2.43 ± 1.57	2.80 ± 2.68	2.27 ± 0.50	0.02
Para-valvular leakage (no.) (%)				0.27
None or trivial	110 (42.8)	36 (44.5)	74 (42.0)	
Mild	144 (56.0)	43 (53.1)	101 (57.4)	
Moderate/Severe	3 (1.2)	2 (2.5)	1 (0.6)	
Laboratory data before discharge				
Hemoglobin (lowest) (g/dL)	9.6 ± 1.5	10.1 ± 1.4	9.3 ± 1.4	<0.01
Platelet (lowest) (x10^5^/μL)	10.0 ± 4.4	10.2 ± 4.9	9.9 ± 4.2	0.92
BNP (n = 213) (pg/mL)	183 ± 183	120 ± 120	216 ±201	<0.01
Medications at discharge (no.) (%)				0.04
SAPT or DOAC alone	37 (14.3)	6 (7.4)	31 (17.5)	
DAPT	183 (70.9)	67 (82.7)	116 (65.5)	
SAPT + DOAC	34 (13.2)	7 (8.6)	27 (15.3)	
Others	4 (1.6)	1 (1.2)	3 (1.7)	
Long-term outcomes (no.) (%)				
All cause death	41 (15.9)	3 (3.7)	38 (21.5)	<0.01
Any coronary revascularization	2 (0.8)	1 (1.2)	1 (0.6)	0.53
MACCE	57 (22.1)	8 (9.9)	49 (27.7)	<0.01
Cardiovascular death	27 (10.5)	1 (1.2)	26 (14.7)	<0.01
Hospitalization of ADHF	22 (8.5)	2 (2.5)	20 (11.3)	0.02
Disabling stroke	15 (5.8)	5 (6.2)	10 (5.6)	1.00

TAVI, trans-catheter aortic valve implantation; LVEF, left ventricular ejection fraction; BNP, brain natriuretic peptide; SAPT, single anti-platelet therapy; DAPT, dual anti-platelet therapy; DOAC, direct oral anti-coagulant; MACCE, major adverse cardiovascular or cerebrovascular events: Composite of cardiovascular death, history of hospitalization due to acute de-compensated heart failure (ADHF), and disabling stroke.

Table 4 shows the results of multivariate COX hazard analysis. The initial model included elderly, history of AF, history of AV blocks, value of albumin, eGFR, anemia determined by WHO criteria, moderate or severe PH, diuretics users, STS score and AS detected by heart murmur as independent variables. The AS patients primarily diagnosed by heart murmur was inversely associated with MACCE (HR 0.38, 95%CI 0.17–0.86, P = 0.020). Elderly (age ≥90 year-old) (HR 1.83, 95%CI 1.00–3.35, P = 0.049), history of AF (HR 2.67, 95%CI 1.53–4.66, P = 0.001), and lower albumin (<3.5 g/dL) (HR 1.92, 95%CI 1.04–3.55, P = 0.036) were significantly associated with MACCE.

**Table 4:** Multivariate COX hazard analysis.

Dependent variables: “MACCE”	HR	95%CI	P value
AS detected by heart murmur	0.38	0.17–0.86	0.020
Elderly (≥90 year-old)	1.83	1.00–3.35	0.049
History of AF	2.67	1.53–4.66	0.001
Albumin (<3.5 g/dL)	1.92	1.04–3.55	0.036

MACCE, major adverse cardiovascular or cerebrovascular events: Composite of cardiovascular death, history of hospitalization due to acute de-compensated heart failure, and disabling stroke; AS, aortic valve stenosis, AF, atrial fibrillation.

**Fig 3:**
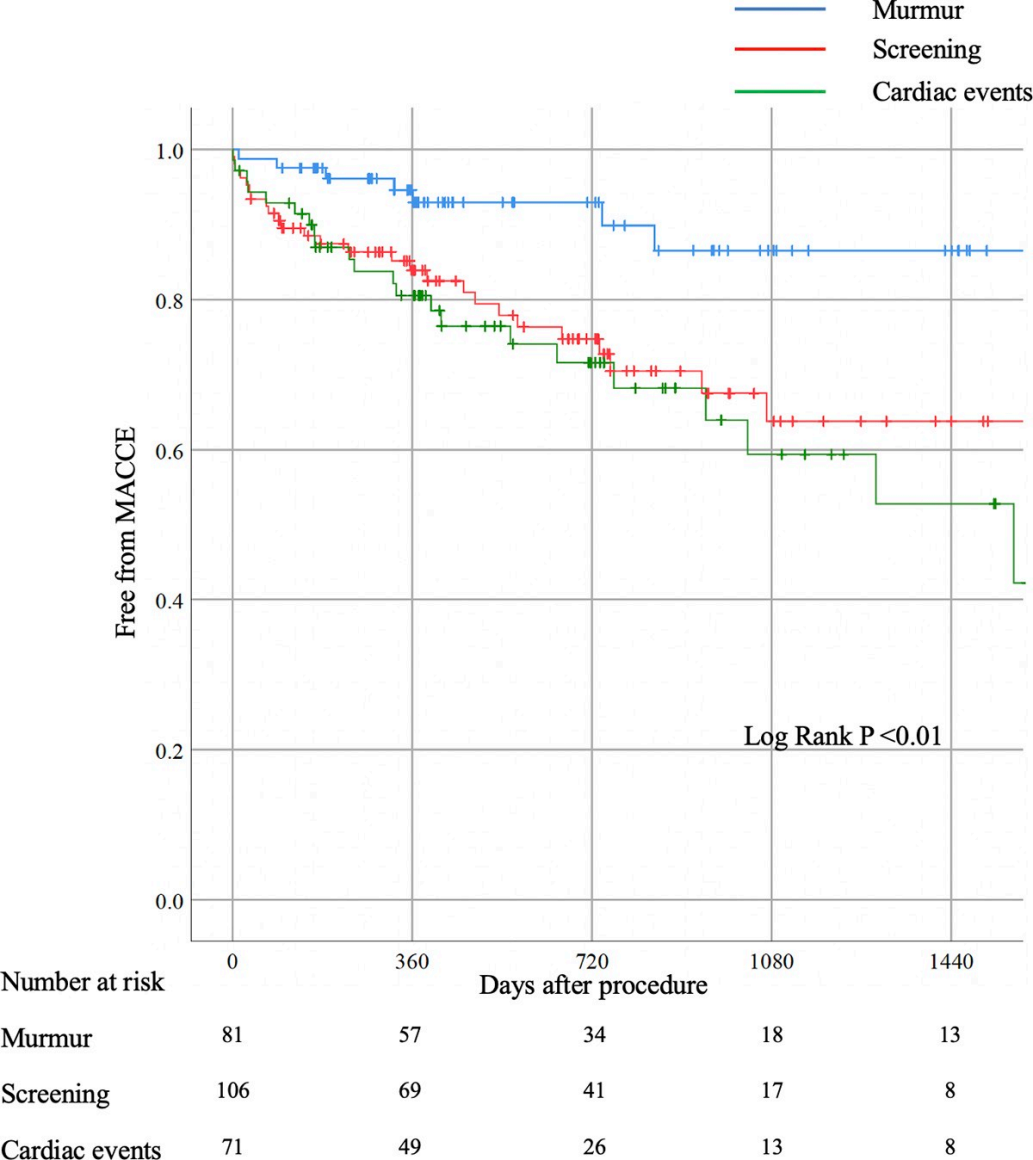
Kaplan-Meyer survival curve analysis among three groups. Each group defined according to the primary reasons like below; Murmur group: Equal the murmur group. Screening group: Composite of screening for cardiac symptom and non-cardiac pre-operations. Cardiac events group: hospitalization for some cardiovascular events.

Furthermore, since the other-reason group included two different types of patients, the other-reason group was further divided into the following two groups: one was the screening group, defined as patients underwent screening echocardiography because of any cardiac symptom or non-cardiac surgery, and the other was the cardiac events group, defined as patients underwent echocardiography after having cardiovascular events. Kaplan-Meier survival curves were constructed among the murmur group, screening group, and cardiac events group (Fig 3). MACCE was less frequently observed in the murmur group than in other two groups (P <0.01).

## Discussion

We included 258 AS patients who underwent TAVI, and divided those into the murmur group (n = 81) and the other-reason group (n = 177). Kaplan-Meier curves showed that the incidence of MACCE was significantly less in the murmur group than in the other-reason group. Multivariate COX hazard analysis also showed that the murmur group was inversely associated with MACCE after controlling confounding factors.

We should discuss why the murmur group showed the better prognosis than the other-reasons group did. First, the murmur group included relatively younger patients, and had better values of albumin, eGFR, hemoglobin, and BNP as compared with the other-reason group. Furthermore, STS score and logistic EuroSCORE were also better in the murmur group than in the other-reason group. Thus, the murmur group was consisted of the patients who had less background diseases. Second, the other-reason group might include many AS patients with severe symptoms, whereas the murmur group might include many AS patients with mild or slight symptoms. In fact, the other-reason group included 79 patients with some cardiovascular symptoms and 71 patients who were hospitalized for cardiovascular events, which accounted for 85% of the other-reason group. As compared to asymptomatic AS patients, symptomatic AS patients would have worse clinical outcomes [10,11]. Third, the other-reason group might include more patients who were suffering from malignant diseases. Fig 1 shows that there were at least 11 patients who had active malignancy in the other-reason group. On the other hand, no patients in the murmur group had active malignant diseases at the time of TAVI. Thus, the greater prevalence of active malignant disease might have influenced the long-term outcomes in the other-reason group. Although the deaths directly related to malignancy were counted as non-cardiovascular death [12], patients with active malignancy disease would have the greater risk of cardiovascular events such as pulmonary embolism. Forth, the other-reason group included more preoperative patients for orthopedic diseases, such as hip fractures. Several groups reported that the long-term mortality was greater in patients after lower limb fracture than in the general population [26–28]. Of note, Sathiyakumar et al. reported that the mortality was emphasized by aging and cardiovascular diseases in patients after hip fraction [29].

Elderly has been well known predictors for long-term outcomes related to TAVI because of their limited lifetime or more backgrounds diseases such as malignancy [9,30]. AF has been reported as one of major predictors for MACCE after TAVI [31,32]. Several mechanisms such as structural remodeling, myocardial fibrosis or loss of atrioventricular synchronicity reduced were reported [33,34]. Some articles have also reported the relationship between malnutrition or hypoalbuminemia and MACCE after TAVI [8,9]. Our results were consistent with those literatures.

The present study might shed light on the auscultation to find AS patients. Since the clinical outcomes of the patients primarily diagnosed by heart murmur were better, the importance of regular auscultation by the primary care physicians should be emphasized, especially in asymptomatic elderly patients who had not been diagnosed with AS. It is also important to order echocardiography if the primary care physicians notice heart murmur. Furthermore, even asymptomatic AS patients sometimes have a poor prognosis, especially in the cases of rapid progression, low left ventricular (LV) function, or very severe AS (the max velocity >5 m/s) [11,35–37]. Auscultation would be also important to find those high-risk asymptomatic AS patients.

### Study limitations

The present study has several study limitations. As this study was a retrospective, single-center study, there is a potential selection bias. Since most patients were referred to our medical center from other clinics or hospitals, the initial medical history largely depended on the description of the referral letter, which might not contain sufficient information. Moreover, although the heart murmur of AS is typically systolic ejection murmur, pan-systolic murmur cases were also included in the murmur group, because some medical records did not describe the detailed characteristics of each murmur. Because sample size was limited, the statistical analysis has an inherent risk of beta error [38].

## Conclusions

Among AS patients who underwent TAVI, the patients whose primary reason for AS diagnosis was heart murmur was significantly associated with favorable long-term clinical outcomes.

## Supporting information

S1 Dataset (XLSX)
